# Non-invasive measurement of pulse pressure variation using a finger-cuff method (CNAP system): a validation study in patients having neurosurgery

**DOI:** 10.1007/s10877-021-00669-1

**Published:** 2021-02-25

**Authors:** Moritz Flick, Phillip Hoppe, Jasmin Matin Mehr, Luisa Briesenick, Karim Kouz, Gillis Greiwe, Jürgen Fortin, Bernd Saugel

**Affiliations:** 1 Department of Anesthesiology, Center of Anesthesiology and Intensive Care Medicine, University Medical Center Hamburg-Eppendorf, Martinistrasse 52, 20246 Hamburg, Germany; 2 CNSystems Medizintechnik, Graz, Austria; 3 Outcomes Research Consortium, Cleveland, OH USA

**Keywords:** Hemodynamic monitoring, Fluid responsiveness, Cardiac preload, Dynamic preload variable, Volume clamp method, Vascular unloading technology

## Abstract

The finger-cuff system CNAP (CNSystems Medizintechnik, Graz, Austria) allows non-invasive automated measurement of pulse pressure variation (PPV_CNAP_). We sought to validate the PPV_CNAP_-algorithm and investigate the agreement between PPV_CNAP_ and arterial catheter-derived manually calculated pulse pressure variation (PPV_INV_). This was a prospective method comparison study in patients having neurosurgery. PPV_INV_ was the reference method. We applied the PPV_CNAP_-algorithm to arterial catheter-derived blood pressure waveforms (PPV_INV−CNAP_) and to CNAP finger-cuff-derived blood pressure waveforms (PPV_CNAP_). To validate the PPV_CNAP_-algorithm, we compared PPV_INV−CNAP_ to PPV_INV_. To investigate the clinical performance of PPV_CNAP_, we compared PPV_CNAP_ to PPV_INV_. We used Bland–Altman analysis (absolute agreement), Deming regression, concordance, and Cohen's kappa (predictive agreement for three pulse pressure variation categories). We analyzed 360 measurements from 36 patients. The mean of the differences between PPV_INV−CNAP_ and PPV_INV_ was −0.1% (95% limits of agreement (95%-LoA) −2.5 to 2.3%). Deming regression showed a slope of 0.99 (95% confidence interval (95%-CI) 0.91 to 1.06) and intercept of −0.02 (95%-CI −0.52 to 0.47). The predictive agreement between PPV_INV−CNAP_ and PPV_INV_ was 92% and Cohen’s kappa was 0.79. The mean of the differences between PPV_CNAP_ and PPV_INV_ was −1.0% (95%-LoA−6.3 to 4.3%). Deming regression showed a slope of 0.85 (95%-CI 0.78 to 0.91) and intercept of 0.10 (95%-CI −0.34 to 0.55). The predictive agreement between PPV_CNAP_ and PPV_INV_ was 82% and Cohen’s kappa was 0.48. The PPV_CNAP_-algorithm reliably calculates pulse pressure variation compared to manual offline pulse pressure variation calculation when applied on the same arterial blood pressure waveform. The absolute and predictive agreement between PPV_CNAP_ and PPV_INV_ are moderate.

## Introduction

Pulse pressure variation (PPV) caused by mechanical ventilation can predict fluid responsiveness [1, 2]. PPV is determined by heart-lung interactions; mechanical ventilation with positive airway pressure causes cyclic changes in venous return and cardiac preload resulting in variable changes in the arterial blood pressure waveform that can be quantified by PPV [3, 4]. Automated measurement of PPV requires continuous recording and analysis of the arterial blood pressure waveform, usually invasively using an arterial catheter.

In recent years, innovative finger-cuff technologies became available that allow continuous recording of the arterial blood pressure waveform and PPV calculation in a non-invasive manner [5–10]. The CNAP system (CNAP Monitor 500; CNSystems Medizintechnik, Graz, Austria) is one commercially available finger-cuff system and has been validated for arterial blood pressure and cardiac output measurements [11]. Using a proprietary algorithm, the CNAP system also automatically calculates PPV (PPV_CNAP_).

We here sought to (a) validate the PPV_CNAP_-algorithm and (b) investigate the absolute and predictive agreement between PPV_CNAP_ and arterial catheter-derived manually offline calculated PPV (PPV_INV_).

## Material and methods

### Study design

This was a prospective method comparison study comparing non-invasive finger-cuff-derived with invasive arterial catheter-derived arterial blood pressure as well as PPV in patients having neurosurgery. Here, we report PPV results. The arterial blood pressure results will be reported separately. The study was approved by the ethics committee (Ethikkomission der Ärztekammer Hamburg, Hamburg, Germany; registration number PV6048) and conducted in operating rooms of the University Medical Center Hamburg-Eppendorf between April and October 2019. All patients provided written informed consent.

### Inclusion and exclusion criteria

We included adult patients (≥ 18 years) who were scheduled for neurosurgery and required invasive arterial blood pressure monitoring using an arterial catheter as part of routine care. We excluded patients with vascular implants at the upper extremities, finger oedema, impairment in peripheral perfusion (e.g., Raynaud syndrome, peripheral artery disease, or arterial-venous shunts), cardiac arrhythmia, valvular heart disease grade 2 or above, excessive movement and/or seizures, or cardiac assist devices. For this analysis of PPV only patients with appropriate ventilator settings (tidal volume ≥ 8 mL kg^−1^ predicted body weight, respiratory rate ≥ 10 min^−1^, and positive end-expiratory pressure ≤ 5 cm H_2_O) were included.

### Automated PPV_CNAP_-algorithm

**Fig. 1:**
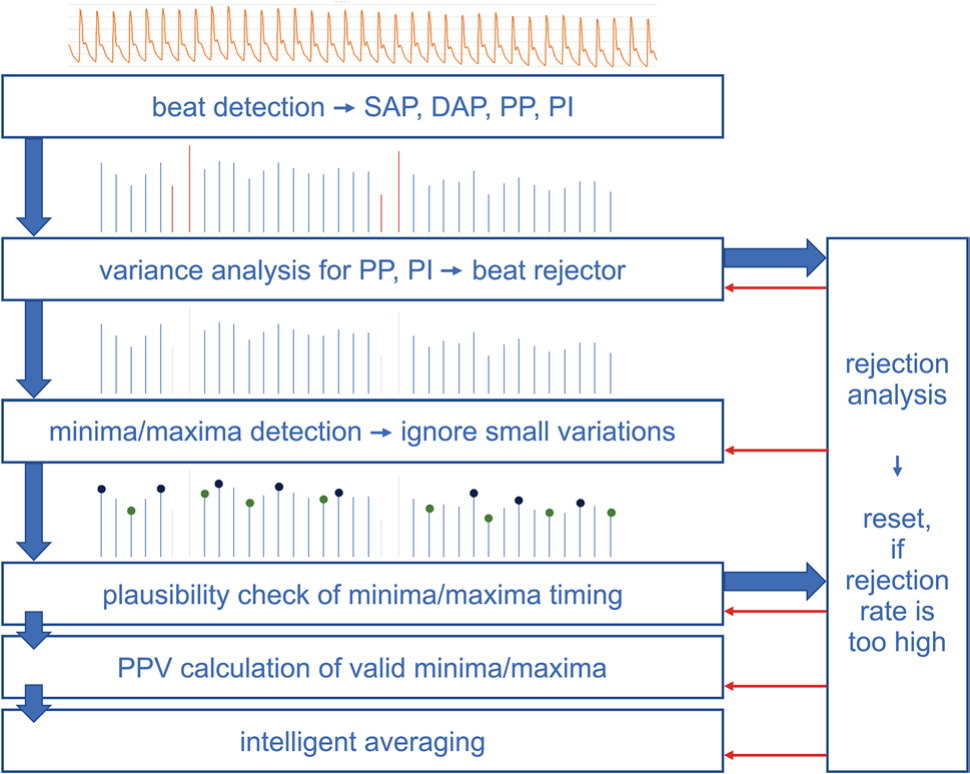
Schematic illustration of the PPV_CNAP_-algorithm. *SAP* systolic arterial blood pressure, *DAP* diastolic arterial blood pressure, *PP* pulse pressure, *PI* pulse interval, *PPV* pulse pressure variation

The PPV_CNAP_-algorithm is a computer algorithm to detect and analyze ventilation-induced swings in the arterial blood pressure waveform and automatically calculate PPV as illustrated in Fig. 1. In short, the PPV_CNAP_-algorithm applies an adapted beat detection algorithm [12] to the arterial blood pressure waveform to obtain systolic and diastolic arterial blood pressure, pulse pressure (PP), and pulse interval (PI). PP and PI are compared to the average PP and PI of previous heart beats. If the difference between a new PP or PI value and their average values of previous heart beats exceeds a certain threshold, the beat could be a premature beat and is therefore excluded from further calculation. The tolerance level is adaptively adjusted by the variance of PP and PI. Next, a PP minimum-maximum detector is applied to the time series of PP values. The detector is made somewhat “fuzzy” to ignore small variations in the PP series. The time appearance of minimum PP (PP_min_) and maximum PP (PP_max_) undergo a plausibility check by using the average of previous verified PP_min_ and PP_max_. After verification, PPV is calculated as: PPV = 200 × (PP_max_ – PP_min_)/(PP_max_ + PP_min_) (%).

Note that one PP_max_/PP_min_ pair corresponds to a half of a respiratory cycle. PPV_CNAP_ is calculated by averaging six PP_max_/PP_min_ pairs corresponding to the last three respiratory cycles. Additionally, outlier detection is used; PPV values higher than 40% are completely rejected. Further, if the difference between a new PPV value and the previous one is higher than a certain threshold, this new PPV value is used for calculation only with a 50% weight. If the PPV value is confirmed by the next measurement, the PPV value is then considered with a full 100% weight.

If plausibility checks of new beats or new PP_min_ or PP_max_ fail too often, all average and variance variables are reset. The PPV_CNAP_-algorithm is newly initialized and re-starts the calculation of average and variance variables from scratch.

### PPV measurements

After induction of general anesthesia, all patients were ventilated with a tidal volume of 8 mL kg^−1^ predicted body weight, a respiratory rate of ≥ 10 min^−1^ adjusted to end-expiratory carbon dioxide, and a positive end-expiratory pressure of ≤ 5 cm H_2_O. After insertion of the radial arterial catheter, the CNAP system’s upper-arm-cuff was attached on the ipsilateral arm. The CNAP finger-cuff was placed on the index and middle finger of the contralateral arm. CNAP finger-cuff arterial blood pressure measurements were calibrated to oscillometric arterial blood pressure measurements every 30 min in the first 27 patients. We changed this to the maximal calibration interval of 60 min during the study and calibrated every 60 min in the last 9 patients. Arterial blood pressure recording was started after positioning of the patient in the operating room and continued until the end of surgery. The continuous arterial blood pressure waveforms measured non-invasively with the CNAP system and invasively with the arterial catheter were simultaneously displayed and recorded on the patient monitor (Infinity Delta Monitor; Dräger, Lübeck, Germany). Both waveforms were extracted to a personal computer (eData Data Grabber; Dräger) and beat-to-beat measurements were used for further offline analysis.

We randomly selected 10 60-s episodes of each patient. Within these episodes, we identified a period with at least three visible swings in PP in the non-invasive and invasive arterial waveform, which were used for further analysis.

We calculated PPV_INV−CNAP_ by applying the PPV_CNAP_-algorithm to the arterial blood pressure waveform recorded invasively using the arterial catheter. PPV_CNAP_ was automatically calculated using the PPV_CNAP_-algorithm based on the arterial blood pressure waveform recorded non-invasively with the CNAP system. PPV_INV_ was calculated manually from the arterial blood pressure waveform recorded invasively using the arterial catheter (reference method).

### Statistical analysis

Descriptive data are reported as mean ± standard deviation (SD) for continuous data and as absolute frequency and percentage for categorical data.

Using Bland–Altman analysis accounting for repeated measurements within individuals [13, 14], we compared (a) PPV_INV−CNAP_ and PPV_INV_ to validate the PPV_CNAP_-algorithm per se and (b) PPV_CNAP_ and PPV_INV_ to investigate the absolute agreement between PPV_CNAP_ and PPV_INV_. For each comparison, we calculated the mean of the differences between the two methods (test method minus reference method), the SD of the mean of the differences, and the 95% limits of agreement (95%-LoA; i.e., mean of the differences ± 1.96 SD of the mean of the differences) and the 95% confidence intervals (95%-CI) around the 95%-LoA to quantify the trueness and precision of agreement [15, 16]. We additionally describe the correlation between PPV_INV−CNAP_ and PPV_INV_ and between PPV_CNAP_ and PPV_INV_ by Deming regression for scattered plots with 95%-CI [17, 18]. We assessed the predictive agreement for fluid responsiveness across three predefined categories (PPV < 9%, PPV 9 to 13%, PPV > 13%) between PPV_CNAP_ and PPV_INV_. These PPV categories reflect PPV thresholds used for clinical decision making regarding fluid therapy in clinical practice [19, 20]. The predictive agreement across these three categories was calculated as the number of concordant paired measurements divided by the total number of paired measurements. In addition, we calculated Cohen’s kappa [21]. A Cohen’s kappa of < 0 indicates no agreement, 0–0.20 slight, 0.21–0.40 fair, 0.41–0.60 moderate, 0.61–0.80 substantial, and 0.81–1.00 almost perfect agreement. Statistical analyses were performed using Microsoft Excel (Microsoft, Redmond, WA, USA), SPSS 25 (IBM, Armonk, NY, USA), and Matlab (The MathWorks, Natick, MA, USA).

## Results

### Study cohort

**Fig. 2:**
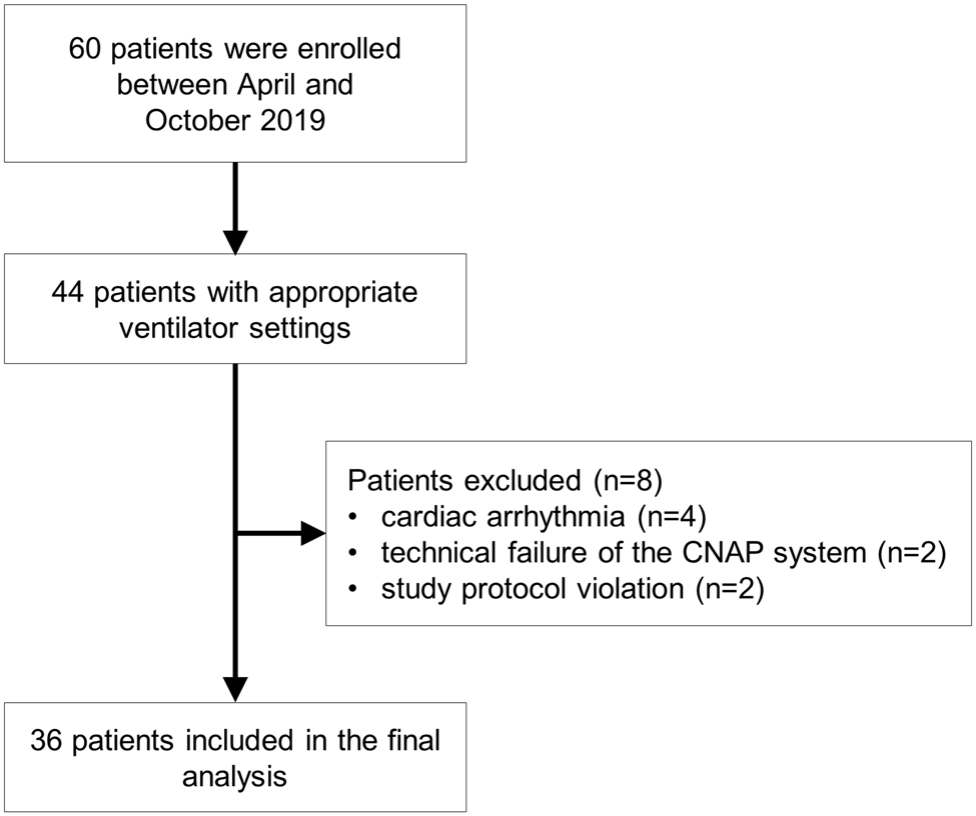
Flow chart illustrating patient enrollment and reasons for exclusion.

A total of 44 patients were available for this analysis, but eight were excluded. We excluded four patients due to cardiac arrhythmia, two patients because of technical failure of the CNAP system, and two patients because of study protocol violations (Fig. 2). We thus included 36 patients with a total of 360 measurements in the final analysis. Patient characteristics are presented in Table 1.

**Table 1:** Patient characteristics

Sex female	18 (50)
Age, years	53.4 ± 13.6
Height, cm	173.3 ± 8.4
Weight, kg	80.9 ± 19.1
ASA class I/II/III/IV, n	3/20/12/1
Duration of measurement, min	167.5 ± 59.3
Type of surgery	
Intracranial tumor resection	24 (66.7)
Aneurysm repair surgery	8 (22.2)
Intracranial biopsy	1 (2.8)
Cranial fracture	1 (2.8)
Hippocampectomy	1 (2.8)
Cervical spine surgery	1 (2.8)

Data are shown as mean ± standard deviation or absolute numbers (percentages)

*ASA class* American Society of Anesthesiologists Physical Status class

### Validation of the PPV_CNAP_-algorithm

**Fig. 3:**
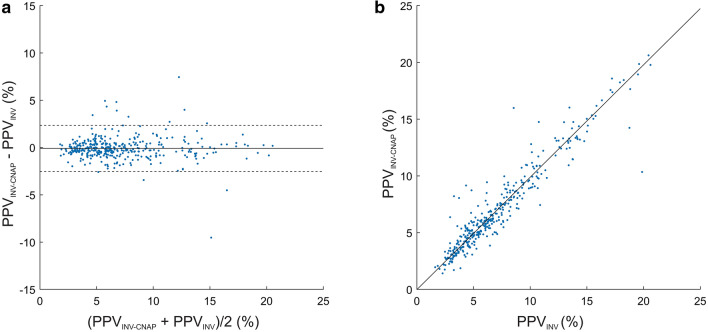
Bland–Altman and scatter plot comparing PPV_INV−CNAP_ and PPV_INV_. **a** Bland–Altman plot illustrating the mean of the differences (bold horizontal line) and 95% limits of agreement (lower and upper dashed horizontal lines) between PPV_INV−CNAP_ and PPV_INV_. **b** Scatter plot with Deming regression (bold line) illustrating the relation of PPV_INV−CNAP_ and PPV_INV_. *PPV*_INV−CNAP_ arterial catheter-derived automatically calculated pulse pressure variation using the PPV_CNAP_-algorithm, *PPV*_INV_ arterial catheter-derived manually calculated pulse pressure variation

The mean of the differences ± SD between PPV_INV−CNAP_ and PPV_INV_ was −0.1 ± 1.2% (95%-LoA −2.5 to 2.3%) (Fig. 3, Table 2). For the comparison between PPV_INV−CNAP_ and PPV_INV_, the Deming regression showed a slope of 0.99 (95%-CI 0.91 to 1.06) and an intercept of −0.02 (95%-CI −0.52 to 0.47) (Fig. 3). The predictive agreement for fluid responsiveness between PPV_INV−CNAP_ and PPV_INV_ was 92% with a Cohen’s kappa of 0.79 (Table 3).

**Table 2:** Absolute and predictive agreement between PPV_INV−CNAP_ vs. PPV_INV_ and PPV_CNAP_ vs. PPV_INV_

	Mean of the differences (%)	SD of the mean of the differences (%)	Lower 95%-LoA (95%-CI) (%)	Upper 95%-LoA (95%-CI) (%)	Deming regression	Predictive agreement (%)	Cohen's kappa
PPV_INV−CNAP_ vs. PPV_INV_	−0.1	1.2	−2.5 (−2.7 to −2.3)	2.3 (2.2 to 2.5)	−0.02 + 0.99x	92	0.79
PPV_CNAP_ vs. PPV_INV_	−1.0	2.7	−6.3 (−6.7 to -5.9)	4.3 (3.9 to 4.7)	0.10 + 0.85x	82	0.48

*PPV*_*INV*_ arterial catheter-derived manually calculated pulse pressure variation, *PPV*_*INV−CNAP*_ arterial catheter-derived automatically calculated pulse pressure variation using the PPV_CNAP_-algorithm, *PPV*_*CNAP*_ CNAP finger-cuff-derived automatically calculated pulse pressure variation using the PPV_CNAP_-algorithm, *SD* standard deviation, *LoA* limits of agreement, *CI* confidence interval

**Table 3:**
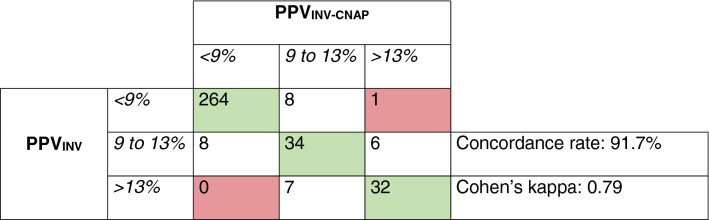
Predictive agreement of pulse pressure variation measurements across the three predefined categories

*PPV*_*INV*_ arterial catheter-derived manually calculated pulse pressure variation, *PPV*_*INV−CNAP*_ arterial catheter-derived automatically calculated pulse pressure variation using the PPV_CNAP_-algorithm

### Agreement between PPV_CNAP_ and PPV_INV_

**Fig. 4:**
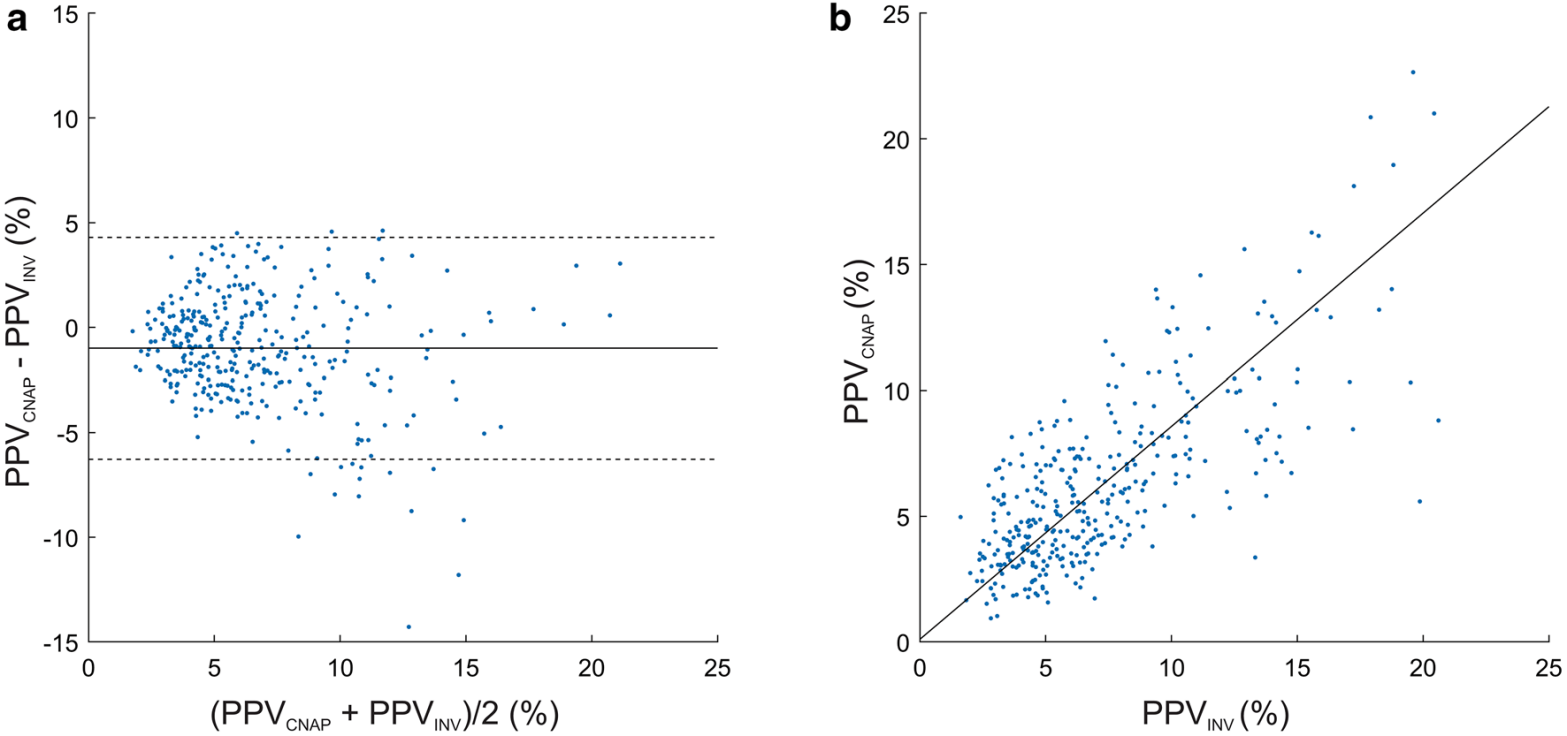
Bland–Altman and scatter plot comparing PPV_CNAP_ and PPV_INV_. **a** Bland–Altman plot illustrating the mean of the differences (bold horizontal line) and 95% limits of agreement (lower and upper dashed horizontal lines) between PPV_CNAP_ and PPV_INV_. **b** Scatter plot with Deming regression (bold line) illustrating the relation of PPV_CNAP_ and PPV_INV_. *PPV*_CNAP_ CNAP finger-cuf-derived automatically calculated pulse pressure variation using the PPV_CNAP_-algorithm, *PPV*_INV_ arterial catheter-derived manually calculated pulse pressure variation

The mean of the differences ± SD between PPV_CNAP_ and PPV_INV_ was −1.0 ± 2.7% (95%-LoA −6.3 to 4.3%) (Fig. 4, Table 2). The Deming regression for the correlation between PPV_CNAP_ and PPV_INV_ showed a slope of 0.85 (95%-CI 0.78 to 0.91) and an intercept of 0.10 (95%-CI −0.34 to 0.55) (Fig. 4). The predictive agreement for fluid responsiveness between PPV_CNAP_ and PPV_INV_ was 82% with a Cohen’s kappa of 0.48 (Table 4).

**Table 4:**
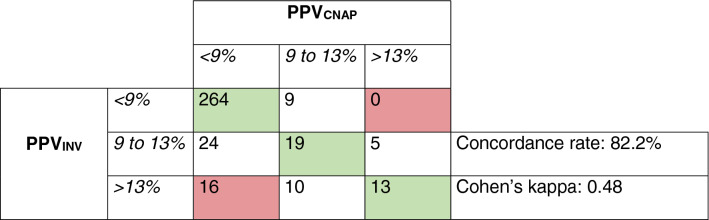
Predictive agreement of pulse pressure variation measurements across the three predefined categories

*PPV*_*INV*_ arterial catheter-derived manually calculated pulse pressure variation, *PPV*_*CNAP*_ CNAP finger-cuff-derived automatically calculated pulse pressure variation using the PPV_CNAP_-algorithm

## Discussion

In this prospective method comparison study, we aimed to validate the PPV_CNAP_-algorithm and investigate the absolute and predictive agreement between PPV_CNAP_ and PPV_INV_ in patients having neurosurgery.

To validate the PPV_CNAP_-algorithm per se (independent from waveform recording), we applied the PPV_CNAP_-algorithm to the arterial blood pressure waveform recorded invasively using an arterial catheter. The absolute agreement—i.e., the trueness and precision of agreement [15, 16]—between PPV_INV−CNAP_ and the manually calculated PPV_INV_ was high. The Deming regression analysis showed a significant correlation between PPV_INV−CNAP_ and PPV_INV_ and the predictive agreement was substantial according to Cohen’s kappa. Our results suggest that the PPV_CNAP_-algorithm reliably calculates PPV and that its measurements are interchangeable with the reference method—the manual offline calculation of PPV—when applied on the same arterial blood pressure waveform.

As a next step, we compared PPV_CNAP_ to the reference PPV_INV_. The absolute agreement between PPV_CNAP_ and PPV_INV_ was lower than between PPV_INV−CNAP_ and PPV_INV_ and the Deming regression indicated a minor proportional difference between the methods. Nonetheless, the predictive agreement between PPV_CNAP_ and PPV_INV_ was moderate according to Cohen’s kappa.

In this study, we used arterial catheter-derived manually calculated PPV (PPV_INV_) as the reference method. There are no consensus guidelines on how to perform PPV validation studies and interpret their results. Specifically, it remains undefined what constitutes clinically acceptable PPV measurement performance. The absolute agreement between PPV_CNAP_ and PPV_INV_ was similar compared with previous studies evaluating the measurement performance of PPV_CNAP_ in critically ill patients [22, 23] and patients having major open abdominal [24] or vascular surgery [25]. A pilot study in only 10 critically ill patients revealed a mean of the differences between PPV_CNAP_ and arterial catheter-derived manually calculated PPV of −2.1% with 95%-LoA of −8.3 to 4.1% [22]. However, the study also included patients who were ventilated with tidal volumes less than 8 mL kg^-1^, which were excluded in our study. In a cohort of 47 critically ill patients with acute circulatory failure, the mean of the differences between PPV_CNAP_ and PPV calculated manually from a femoral arterial blood pressure waveform was −0.6% with 95%-LoA of −6.3 to 5.2% [23]. The authors excluded 17% of patients because the CNAP system was unable to properly record the non-invasive arterial blood pressure waveform [23]. In contrast, we were able to record an arterial blood pressure waveform with the CNAP system in all patients. Our results are in line with a previous study in 35 patients having vascular surgery which showed similar moderate absolute agreement between PPV_CNAP_ and arterial catheter-derived manually calculated PPV before and after volume expansion [25]. Even though our results are in line with previous findings, it is challenging to interpret the absolute agreement of PPV_CNAP_ with PPV_INV_ as no clearly defined thresholds for clinically acceptable PPV differences exist.

When investigating non-invasively measured dynamic cardiac preload variables, their predictive capabilities regarding the prediction of fluid responsiveness may even be more important than absolute agreement with invasive reference measurements. In the before-mentioned study in vascular surgery patients, volume expansion was performed to investigate the ability of PPV_CNAP_ to predict fluid responsiveness. PPV_CNAP_ predicted fluid responsiveness very well according to receiver operating characteristics curve analysis [25]. This was also shown in other studies directly testing the capabilities of PPV_CNAP_ to predict fluid responsiveness, i.e., an increase in cardiac output after a fluid challenge. PPV_CNAP_ and PPV calculated from an invasive arterial blood pressure waveform seem to have similar predictive value [23, 24]. We did not perform a fluid challenge or passive leg-raising test to directly test how well PPV_CNAP_ predicts fluid responsiveness. Instead, we categorized PPV measurements considering clinical decision making based on predefined PPV thresholds for the prediction of fluid responsiveness [19]. PPV_CNAP_ measurements falling in the same category as the respective PPV_INV_ values would subsequently lead to the same decision regarding fluid therapy. The predictive agreement between PPV_CNAP_ and PPV_INV_ across the three categories was over 90% and Cohen’s kappa indicated a substantial predictive agreement. In line with the results of Bland–Altman analysis, the predictive agreement between PPV_CNAP_ and PPV_INV_ was slightly lower, but still over 80% and Cohen’s kappa indicated moderate agreement.

We did not perform preload-changing interventions such as a fluid challenge or passive leg-raising test to assess fluid responsiveness. Nevertheless, we analyzed the agreement between the test and the reference method stratified by different PPV categories according to clinically established thresholds [19]. Data pairs were selected randomly, but data selection bias cannot be ruled out definitely. We did not perform an a priori sample size calculation. Narrow 95%-CI around the 95%-LoA of the means of the differences between PPV_INV−CNAP_ and PPV_INV_ as well as PPV_CNAP_ and PPV_INV_ suggest that the sample size was sufficient though. Additionally, the change of the calibration interval for the CNAP system during the study may have affected the results. We only included patients having neurosurgery and the results can thus not be generalized to other—especially critically ill—patients.

In conclusion, the PPV_CNAP_-algorithm reliably calculates PPV compared to manual offline PPV calculation when applied on the same arterial blood pressure waveform. The absolute and predictive agreement between PPV_CNAP_ and PPV_INV_ are moderate.
